# Limited Genetic Diversity Preceded Extinction of the Tasmanian Tiger

**DOI:** 10.1371/journal.pone.0035433

**Published:** 2012-04-18

**Authors:** Brandon R. Menzies, Marilyn B. Renfree, Thomas Heider, Frieder Mayer, Thomas B. Hildebrandt, Andrew J. Pask

**Affiliations:** 1 Leibniz Institute for Zoo and Wildlife Research, Berlin, Germany; 2 Department of Zoology, the University of Melbourne, Royal Parade, Victoria, Australia; 3 Department of Molecular and Cell Biology, University of Connecticut, Storrs, Connecticut, United States of America; 4 Berlin Museum für Naturkunde, Leibniz Institut für Evolutions- und Biodiversitätsforschung, Invalidenstrasse, Berlin, Germany; Natural History Museum of Denmark, University of Copenhagen, Denmark

## Abstract

The Tasmanian tiger or thylacine was the largest carnivorous marsupial when Europeans first reached Australia. Sadly, the last known thylacine died in captivity in 1936. A recent analysis of the genome of the closely related and extant Tasmanian devil demonstrated limited genetic diversity between individuals. While a similar lack of diversity has been reported for the thylacine, this analysis was based on just two individuals. Here we report the sequencing of an additional 12 museum-archived specimens collected between 102 and 159 years ago. We examined a portion of the mitochondrial DNA hyper-variable control region and determined that all sequences were on average 99.5% identical at the nucleotide level. As a measure of accuracy we also sequenced mitochondrial DNA from a mother and two offspring. As expected, these samples were found to be 100% identical, validating our methods. We also used 454 sequencing to reconstruct 2.1 kilobases of the mitochondrial genome, which shared 99.91% identity with the two complete thylacine mitochondrial genomes published previously. Our thylacine genomic data also contained three highly divergent putative nuclear mitochondrial sequences, which grouped phylogenetically with the published thylacine mitochondrial homologs but contained 100-fold more polymorphisms than the conserved fragments. Together, our data suggest that the thylacine population in Tasmania had limited genetic diversity prior to its extinction, possibly as a result of their geographic isolation from mainland Australia approximately 10,000 years ago.

## Introduction

**Figure 1:**
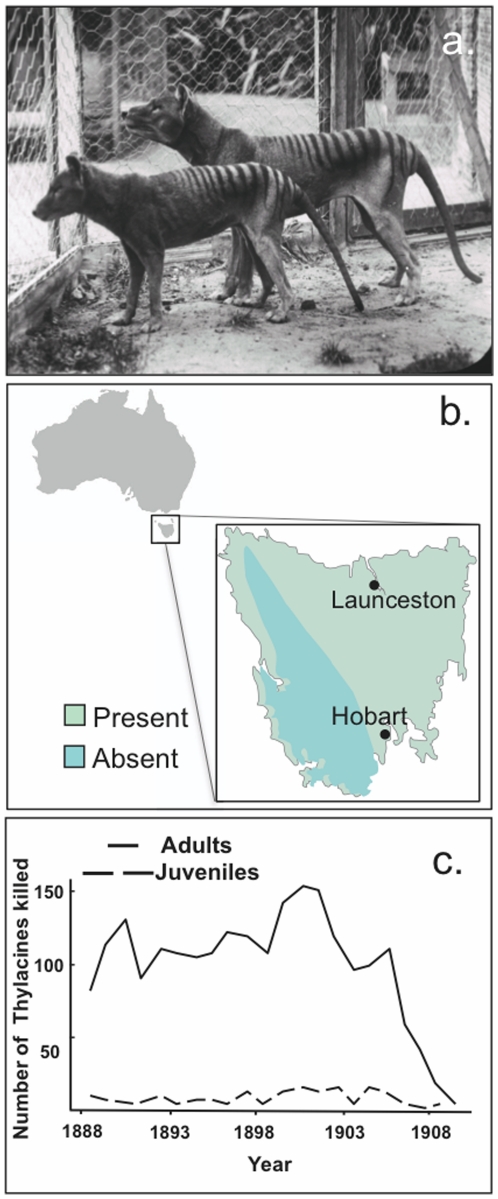
Appearance, bounty collection data and former distribution of the Tasmanian tiger. **a**) Two adult male thylacines, Beaumaris Zoo, Hobart, 1911. **b**) Estimated former range of the Tasmanian tiger based on bounties presented for adult and juvenile individuals. **c**) Number of adult and juvenile thylacines collected during the bounty scheme from 1888–1924. Figures b and c redrawn from (17).

The Tasmanian tiger, *Thylacinus cynocephalus*, is one of the most fascinating animals to have ever lived. A marsupial that evolved in Australasia and was later isolated on the island of Tasmania, it is one of the most striking examples of convergent evolution and appears almost identical, both morphologically and behaviourally, to its eutherian mammal counterpart, the dog or wolf (Figure 1a). Despite their 160-million-year divergence [1], its size, body structure and dentition make it almost indistinguishable from the dog. The presence of a pouch in female thylacines and the posterior positioning of the penis relative to the testes in males were the only external features to distinguish it as having evolved from a marsupial ancestor.

Once found on mainland Australia, a subpopulation of thylacines became isolated on the island of Tasmania after the flooding of Bass Straight approximately 10–13 thousand years ago, and so they avoided the decline and eventual extinction of the mainland population that coincided with the arrival of the dingo, *Canis lupus dingo*, 5–6 thousand years ago [2]. Based on limited observations and bounty information, the thylacine appeared to be a solitary ambush predator that preferred open woodland habitat. Its former range included the north-western, central, eastern and south-eastern parts of the island of Tasmania, but not the mountainous south-west (Figure 1b) [3], [4].

Far from being appreciated, European settlers deemed the thylacine a threat to the developing colonial sheep industry and it was aggressively targeted for eradication by the government with a £1 bounty paid for every animal killed [3]. Mothers with pouch young or live specimens could be sold to zoos or museums for even greater remuneration. As a result, the remaining population was rapidly exterminated from Tasmania during the bounty period from 1888 to 1912, over which time 2,184 specimens were presented for reward (Figure 1c) [3]. The last known wild thylacine was killed in 1930 and the last known animal died in the Hobart Zoo in Tasmania on the 7^th^ of September 1936 [4].

As a testament to its notoriety and uniqueness, there are numerous thylacine specimens in museum collections around the world. Due to their relatively recent extinction, DNA (albeit highly fragmented) can be reliably obtained from soft tissue, pelt and bone samples [5]–[7]. Recently, the sequence was determined for the complete mitochondrial genome of two thylacines (both collected during the bounty; one culled 1893 and the other in 1905) [7]. Surprisingly, these two specimens differed by only 5 nucleotides over almost 16 kilobases of sequence and these differences were outside the hyper-variable region, commonly used as an indicator of genetic variability.

The Tasmanian devil, *Sarcophilus harrisii*, is a closely related and extant carnivorous marsupial that, together with the thylacine, became isolated on the island of Tasmania. The devil population is currently under threat of extinction from a highly contagious and aggressive facial tumor disease [8]. There is currently very limited genetic diversity in the devil population as determined using a range of techniques including microsatellite, major histocompatability complex and whole-genome sequence analyses [8]–[10]. This lack of genetic diversity is thought to have contributed to the rapid spread of the facial tumor disease.

Since the Tasmanian tiger population underwent the same geographic isolation from mainland Australia as the Tasmanian devil, we might also expect to see limited genetic diversity within the thylacine population. This prediction is supported by the study from Miller et al [7] and suggests that remnant populations of Tasmania's marsupial carnivores may have experienced a decline in genetic variability. To resolve whether the genetic paucity described previously for two thylacine specimens was widespread in the Tasmanian population, we sequenced a portion of the hyper-variable region (HVR1) in the mitochondrial DNA (mtDNA) control region from an additional 12 specimens (Table 1). We included the two published sequences [7] in our analysis bringing the total number of thylacines analysed to 14. Here we describe indices of population diversity for the thylacine and compare these results to other populations of marsupial and eutherian carnivores.

**Table 1:** Thylacine specimens sampled and the number of polymorphisms identified in each individual.

Specimen accession #	Sequence Length	Institution	Date collected	Sex	Tissue	Polymorphic Sites
A6.7/3	187	University Museum of Zoology Cambridge	1871	Male	Dried tendon	1
A6.7/5	117	University Museum of Zoology Cambridge	1871	Female	Dried tendon	1
SMF-15682	187	Forschungsinstitut Senckenberg	1852	Male	Pelt	1
HLMD-1241	187	Hessisches Landesmuseum Darmstadt	1887	Female	Pelt	1
HLMD-1249	117	Hessisches Landesmuseum Darmstadt	1888	Unknown	Dried tendon	1
C-3149	187	Museum Victoria	Unknown	Male	Pelt	0
C-5748	187	Museum Victoria	1909	Female	Pelt	0
C-5755	187	Museum Victoria	1909	Pouch young	Fixed tissue	0
C-5756	187	Museum Victoria	1909	Pouch young	Fixed tissue	0
36877	117	Berlin Museum für Naturkunde	Unknown	Male	Dried tendon	1
2986	117	Berlin Museum für Naturkunde	1864	Male	Dried tendon	0
SMNS-2019	117	Staatliches Museum für Naturkunde Stuttgart	1889	Male	Bone	0
I/4691	117	Zoologisches Museum Greifswald	1885	Male	Pelt	0

## Results

### Control region variability

We successfully isolated DNA from all 12 specimens including fixed soft tissues, bone and pelt (Table 1). DNA was typically fragmented with an average size of approximately 100 base pairs (bp). The hyper-variable region of the thylacine mtDNA contains a 72–75 bp repeat in triplicate. As the DNA samples were already considerably fragmented, amplification of the repeat using small overlapping amplicons would not clarify their relative position. Therefore, we amplified a small unique portion of the mtDNA control region directly after the repeat (Figure S1).

We were able to compare six individuals over 187 bp and 12 individuals over 117 bp of the mitochondrial hyper-variable region. There were only six nucleotide differences between all of the animals sequenced, and all of the polymorphisms were within the smaller 117 bp region. These polymorphisms were found in five individuals collected in 1852 (1 difference), 1871 (2 differences), 1887 (1 difference), 1888 (1 difference), and one of unknown collection date (1 difference). Genetic diversity among the specimens, or the chance of two randomly chosen sequences being different, was 60%±21.5% and 68±14% for the 187 and 117 bp fragments respectively. Nucleotide diversity between individual homologous nucleotides was 0.5%±0.47% and 0.8%±0.6% for the 187 and 117 bp fragments respectively, while the mean number of nucleotide differences between sequences was 1.0±0.77 and 1.0±0.72 for the 187 and 117 bp fragments respectively. To determine our expected error rate caused by extrinsic damage to the archived specimen's DNA we sequenced the haplotypes of two offspring derived from a single female specimen. The young were 100% identical to the mother over 187 bp of sequence, suggesting a very low extrinsic DNA damage rate (pouch young were not included in the population analysis).

### Partial mtDNA genome reconstruction

**Figure 2:**
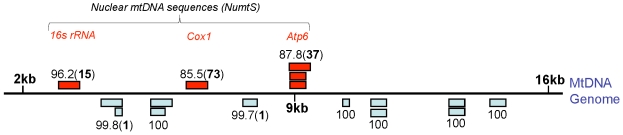
Genomic alignment of 454 reads with the published thylacine mtDNA genome. The 454 generated sequences produced 16 individual alignments with the published genome. Eleven of these fragments covered seven regions and were nearly identical (only two base differences over 2.24 kilobases). The other five fragments covered three locations (*ATP6*, *COX1* and *16s rRNA* genes) and were highly divergent from previously published sequences. Therefore, these sequences likely represent NuMts. Numbers above/below fragments indicate the nucleotide similarity to the published thylacine mtDNA genome and total number of polymorphisms in brackets.

Additional mitochondrial genome sequences were isolated from a 454 whole genome shotgun run using DNA derived from pelt (Specimen C-5748; Table 1). The sequences outside of the hyper-variable region included 16 fragments ranging in size from 142–458 bp (Figure 2). Eleven of these sixteen fragments were 99.91% similar to the published genome, which matches previous estimates of mtDNA variability in the thylacine [7]. However, the remaining five fragments spanning three locations were only 89.2% similar and contained numerous polymorphisms relative to the previously published thylacine mtDNA genomes [7].

Three of these polymorphic fragments formed one contiguous block of 301 nucleotides that mapped to a 79 bp portion of the ATP synthase subunit 8 gene (*ATP8*) and a 222 bp portion of the ATP synthase subunit 6 gene (*ATP6*; Figure 2). This block contained 37 polymorphisms that encoded 15 amino acid changes relative to the published genome [7]. The second variable region mapped to the cytochrome oxidase subunit 1 gene (*COX1*) and contained 73 polymorphisms over 458 bp. These polymorphisms encoded 11 amino acid substitutions (Figure S2).

**Figure 3:**
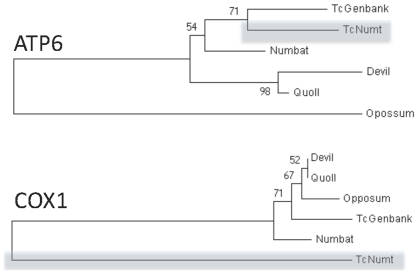
Phylogenetic relationship of the amino acid sequences encoded by the presumptive thylacine *ATP6* and *COX1* NuMts. All of the phylogenetic models grouped the presumptive thylacine COX1 NuMts (TcNumt) separately from both the published thylacine mtDNA genome (TcGenbank) and other marsupials including closely related species and the South American gray short-tailed opossum. However, the ATP8 and ATP6 presumptive NuMts grouped with the published thylacine homolog supported by strong bootstrap values. Trees represent the most conservative estimates of phylogeny attained from using a combination of models (see materials and methods). There were no differences in the groupings for individual genes between models.

We performed phylogenetic analysis of the nucleotide and amino acid (where appropriate) sequences encoded by the variable fragments with closely related marsupial sequences including those from the Tasmanian devil, Northern quoll (*Dasyurus hallucatus*) numbat (*Myrmecobius fasciatus*) and the distantly related South American gray short-tailed opossum (*Monodelphis domestica*). Analysis of the inferred amino acid sequences grouped the thylacine COX1 variable fragment separately from all marsupial orthologs (Figure 3). In contrast, the variable ATP8/ATP6 amino acid sequence grouped with the published thylacine homolog. Phylogenetic analysis of nucleotide sequences for both *ATP8/ATP6* and *COX1* also grouped the variable reads with the published thylacine homologs (Figure S3).

The third variable region mapped to the *16S* ribosomal RNA gene and contained 15 polymorphisms over 389 bp. *16S rRNA* is not translated, thus we cannot provide an amino acid comparison. Phylogenetic analysis also grouped the variable fragment with the published thylacine homolog similar to the *ATP8*/*ATP6* and *COX1* genes (Figure S3). All of the nucleotide phylogenies were supported by high bootstrap values (>91) except for the *COX1* variable nucleotide sequence in which the bootstrap values, but not the groupings, differed between models (ranging from 56–78).

Contamination of our thylacine DNA with bacteria and nucleic acids from other sources was very low (Figure S4). Non-marsupial reads represented less than 10% of our sequences, caused largely by microbial contamination (8.1%). These contaminating reads were removed prior to our analyses.

## Discussion

Our data confirm that the Tasmanian tiger population possessed limited genetic diversity between 1852 and 1909. We only found 6 nucleotide differences altogether in the HVR and seven specimens were identical at all positions. Our genome sequence analysis of a single adult female collected in 1909 also displayed only two nucleotide differences across 2,138 bp (or 0.09%) relative to the mtDNA genomes published previously [7].

The fragments obtained through whole genome shotgun sequencing also included three highly divergent partial sequences of mtDNA genes including *ATP8* and *ATP6*, *COX1* and *16s rRNA*. Several lines of evidence suggest that these variable sequences represent nuclear mitochondrial sequences (NuMts), which are prevalent across multiple taxa from plants to animals and are becoming more readily identified through genome sequencing projects [11], [12]. Firstly, this high level of polymorphism (between 3.8–14.5%) was only observed in these specific fragments. This level of divergence far exceeds that reported for the complete mtDNA coding genomes of many extant mammals including the Tasmanian devil [10] that. Devils possess, on average, only 10 polymorphisms across the entire mtDNA genome excluding the HVR. Secondly, the protein coding portions of these sequences encode numerous amino acid substitutions relative to the published thylacine homologs, and phylogenetic analysis grouped the *COX1* predicted amino acid sequence separately from all other marsupial sequences. The functional *COX1* gene is highly conserved in mammals (Figure S2). Therefore, the 11 amino acid changes seen in the variable sequence suggest that this gene has not been subject to purifying selection. Finally, the possibility of NuMts in the thylacine genome has already been suggested by Miller *et al* [7] based on high levels of variability in the *12s rRNA* and *cytochrome b* sequences produced by Krajewski *et al* [5] that were 88.8% and 84.4% similar to the mtDNA genome produced by 454 sequencing. The Krajewski *et al* [5] differences encoded 33 amino acid substitutions in total.

The phylogenetic grouping of the *ATP8/ATP6* amino acid and nucleotide sequences for the putative NuMts with the published thylacine mtDNA genome, suggests that these sequences are specific to the thylacine and were not incorporated into the nuclear genome before its evolutionary divergence from closely related carnivorous marsupials such as the numbat and Tasmanian devil. The separate phylogenetic grouping of our *COX1* protein from all other marsupial *COX1* proteins can be explained by the high number of polymorphisms (14.5%) relative to its high level of conservation in other marsupials (approximately 97% similar). However, our *COX1* nucleotide sequence still groups phylogenetically with the published thylacine sequence, which suggests that these NuMts are specific to the thylacine lineage (Figure S3).

We have a high level of confidence in the accuracy of our sequence reads since the haplotypes from ethanol-fixed pouch young exactly match that of their mother. These data suggest that thylacine DNA is relatively unaffected by oxidative damage. In addition, the molecular diversity indices for both the 187 and 117 nucleotide comparisons were very similar. The majority of the observed polymorphisms using both Sanger and 454 sequencing methods were not transitions that can result from deamination of ancient DNA. Only one such transition was identified in the control region at position 111 of our Darmstadt specimen relative to the consensus sequence, and this was deemed to be a genuine polymorphism based on repeat observation of sequence variation in multiple clones. Of the two nucleotide differences observed in the highly conserved 454 derived fragments, only one of these could be the product of deamination. Therefore, our estimate of mtDNA genome variability is either 99.91% or 99.96% depending on the inclusion of this one nucleotide. Nevertheless, both estimates fit well with the level of mtDNA variability reported by Miller et al (99.97%) [7].

HVR variability in the thylacine is much lower than that of large outbred carnivore populations such as dogs and wolves that have many polymorphisms over the mtDNA control region as a result of distinct episodes of back-crossing after domestication [13]. Dogs and wolves have an average of 5.3 nucleotide substitutions between individuals, or 2.1% across the hyper-variable region. This is in contrast to our reported 1.0 nucleotide substitutions and 0.5–0.8% variability as seen in our thylacine samples.

The low mtDNA diversity observed in the thylacine may have originated from the geographic isolation of Tasmania from mainland Australia in the recent past. The damaging effect of isolation on the genetic variability of carnivores can be seen in the remnant population of North American wolves (*Canis lupus*), which possess less than half the mtDNA variability of their extinct relatives from the western continental United States [14]. There are relatively few studies of Australian marsupials that compare mitochondrial sequence data within populations because microsatellite data is much more informative for investigations of gene flow and nuclear DNA variability. However, within the remaining population of Leadbeater's possum (*Gymnobelideus leadbeateri*), which occupy small pockets of mountain ash forest in south-eastern Victoria (mainland Australia), nucleotide diversity and mean number of nucleotide differences within sampling groups of eight or more individuals ranged from 0.02–1.2% and 0.12–6.76, respectively [15]. Therefore, our diversity estimates for the thylacine are comparable to some of the least diverse subpopulations of Leadbeater's possum.

Together, our findings suggest that genetic diversity in the Tasmanian tiger population was limited prior to its extinction. Combined with recent data from the Tasmanian devil [10], these studies suggest that the genetic composition of Tasmania's two largest native carnivore populations may have been affected by their geographic isolation from mainland Australia.

## Materials and Methods

### Sampling

Specimens were obtained with permission from the Berlin Museum für Naturkunde, Museum Victoria, University Museum of Zoology Cambridge, Museo Nacional de Ciencias Naturales, Staatliches Museum für Naturkunde Stuttgart, Hessisches Landesmuseum Darmstadt, Übersee Museum Bremen, Forschungsinstitut Senckenberg, University of Heidelberg Museum of Zoology and the Zoologisches Museum Greifswald.

A small sample of pelt tissue or nasal cartilage was cut discretely from individual specimens using a sterile razor blade and gloves. Specimens were kept in sterile cryotubes at room temperature and extracted at the earliest possible convenience. Samples of the alcohol preserved specimens were carefully dissected under sterile conditions. Liver tissue was collected into sterile cryotubes and held at 5°C until extraction.

### DNA extraction

DNA was extracted using the method of Pääbo [16] within a UV-sterilized reverse-flow containment hood, housed in a dedicated clean room separate from the area where PCR amplifications were performed. Bone powder (0.5 g) from one specimen was obtained by using a 1.5 mm drill bit. DNA was extracted by incubation of the bone powder for 48 hours at 42°C in 0.5 M EDTA. This preparation was centrifuged (5 minutes, 15,000 RPM) briefly and new EDTA and 20 µl of 10 mg/ml Proteinase K (Peqlab, Germany) added and incubated for a further 24 hours. The digested sample was centrifuged, 1 ml of supernatant was removed and combined with fresh phenol, mixed thoroughly and again centrifuged. The aqueous fraction was then removed and the process repeated again with fresh phenol, and then once more with chloroform/isoamyl alcohol (24∶1). The final supernatant was removed and 1 ml of ice-cold 100% ethanol added to the solution and incubated at −20°C overnight. This preparation was then centrifuged for 30 mins at 15,000 RPM, the ethanol removed, and the DNA resuspended in 20 µL of ultra clean water.

### PCR amplification

Mitochondrial sequences were amplified and isolated using a two-step PCR method. The first-round PCR was performed as a multiplex reaction in 50 µL total volume using high fidelity, proof reading polymerase master mix (25 µL; Bio-Rad, USA), three primer sets (1 µL each at 10 ng/µL), BSA at a concentration of 1 µg/µL, 1 µL of DNA and ultra sterile water to 50 µL. The conditions for the PCR were 98°C for 3 minutes, 25 cycles of 98°C for 10 seconds, 48°C for 10 seconds, 72°C for 30 seconds and a final extension of 72°C for 5 minutes. The whole PCR sample was then purified using a MinElute column (Qiagen, Germany) and resuspended in 10 µl of elution buffer ready for the second-round PCR. The second PCR was performed using specific primer sets in a 25 µL reaction with 12.5 µL Dream Taq (Thermo Scientific, Germany) or GoTaq (Promega, USA), 1 µL of primer (10 ng/µL), 2 mµ of purified PCR DNA, and water to 25 µL. Amplification conditions were as follows: initial denaturation 95°C for 4 minutes, 30 cycles of 95°C for 30 seconds, 48°C for 30 seconds, 72°C for 1 minute, and a final extension of 72°C for 5 minutes. Primer sets included (Forward primer 1: CTTTACCTAATATTTCTCAT; reverse primer 1: GGATTATGGTCGTAGATCAT; forward primer 2: ACTTACATAGACATTTCATC; reverse primer 2: AAATGACCGAGTTGCTGATC). All sequences were deposited in the Genbank database (accession numbers: JQ318672–JQ318683). No template controls were processed using the same conditions. Final PCR products were separated using electrophoresis on 1% polyacrylamide gels stained with GelRed (Biotrend, Germany). Positive bands were isolated under UV light and purified for sub-cloning.

### Sub-cloning and Sequencing

Positive PCR fragments were sub-cloned into pBluescript or pGEM-T easy (Promega, USA) for sequencing. A minimum of 4 plasmids per PCR fragment were sequenced in both forward and reverse directions using T7 and SP6 primers. A consensus sequence was constructed for each individual based on multiple clones. Nucleotide positions that may indicate deamination sites relative to the sequences published by Miller et al [7] were excluded by performing a secondary PCR from the original DNA. The presence of nuclear mitochondrial sequences (NuMts) was excluded based on overall similarity of the sequences relative to published thylacine mtDNA genomes and the conservation of individual sequences generated from overlapping primer combinations.

### Sequence analysis

Sequences were aligned using Mega version 5.0 [17] and standard molecular indices of diversity were generated using Arlequin version 3.1 [18]. Sequence analysis was performed on 187 bp of mtDNA sequence from 6 specimens (including those published by Miller et al., 2009; Table 1), and 117 bp of mtDNA sequence from 12 specimens. Sequences derived from pouch young were not included in this analysis because of their known relationship to the female specimen. Phylogenetic trees were constructed using Mega version 5. All trees were constructed using bootstrap tests (1000 replicates) and default settings for a range of phylogeny methods including maximum likelihood (Model: Jones-Taylor-Thornton for protein; Tamura-Nei for nucleotide), neighbour joining (Model: p-distance for protein; maximum composite likelihood for nucleotide) and minimum evolution (Model: p-distance for protein; maximum composite likelihood for nucleotide). All of these phylogeny methods produced identical groupings for each of our gene comparisons. Published sequences used for these comparisons included NCBI accession numbers; FJ515780, YP002519811, YP002519812, YP002519809 (Tasmanian tiger), FN666604, CBJ55445, CBJ55446, CBJ55443 (Tasmanian devil), NC007630, YP423966, YP423967, YP423964 (Northern quoll), FN666603, YP002519876, YP002519877, YP002519874 (Numbat) and NC006299, YP087183, YP087184, YP087181 (Opossum).

### 454 sequencing and mapping

DNA was extracted from a thylacine pelt specimen (C5748) as previously described [6]. The total DNA sample (without size selection) was processed for library construction using the random shotgun approach and the GS FLX Titanium library preparation kit (454 Life Sciences) using 14 cycles for library amplification. Genomic library fragments were amplified with the GS FLX Titanium SV emPCR Kit (454 Life Sciences). The subsequent library was sequenced using the GS FLX system (454 Life Sciences).

Our run produced 4,976 reads with an average length of 301 bases. The reads were mapped to the previously published thylacine mitochondrial genomes FJ515780 and FJ515781 [7] using the program BWA 0.6.1 [19]. BWA was able to map 16 reads to each genome. Human contamination was computationally removed by mapping reads to the human genome, version GRCh37.64 from Ensembl, and any that matched were removed from the pool of reads. To evaluate the effectiveness of this, all 4,976 reads were mapped to NCBI's nr database using BLAST [20], [21]. The output was parsed using a custom bioperl script to identify the organism associated with the highest E-value. Reads with more than 50% that mapped to the lowest E-value hit were counted as that organism (Genbank accession numbers: JQ318684–JQ318693). We determined the high quality of our thylacine 454 library by assessing sequence similarity of 2699 uniquely mapping reads (not derived from repeat elements) to the Tasmanian devil genome. These reads showed an average of 98.5% sequence identity, consistent with the evolutionary divergence of these species and previous genomic comparisons.

## Supporting Information

Figure S1 **Position of repetitive element and the amplified region within the thylacine mtDNA CR-1.** The thylacine CR-1 contains a repetitive sequence of 72–75 nucleotides in triplicate. This region was avoided as the organization of small <100 bp fragments could not determined. Instead, we targeted a 187 bp region after the repeat (Broken line box: repetitive section; unbroken line box: sequenced section; Arrows: position of forward and reverse primers).
(TIFF)

Figure S2 **Amino acid alignment of the presumptive ATP8/ATP6 and COX1 NuMts relative to other marsupial homologs.** Our presumptive NuMts for the *ATP8/ATP6* and *COX1* genes encoded 15 and 11 amino acid substitutions relative to the published thylacine homologs, respectively. The COX1 differences contrast much clearer than the ATP8/ATP6 differences as this gene is highly conserved in mammals.
(TIF)

Figure S3 **Phylogenetic relationship of the variable** ***ATP8/ATP6*** **,** ***COX1*** **and** ***16s rRNA*** **nucleotide sequences.** DNA sequence analysis of the variable thylacine sequences (TcNumt) grouped consistently with the sequences from the published thylacine mtDNA genome (TcGenbank) indicating that they likely inserted into the nuclear genome after the divergence of the thylacine from other marsupials. Trees represent the most conservative estimates of parsimony attained from using a combination of models (see materials and methods). There were no differences in the groupings for individual genes between models.
(TIF)

Figure S4 **Quantity of transcripts derived from the thylacine 454 preparation.** The 454 preparation was overwhelmingly composed of thylacine-specific transcripts, which made up 90% of the reads. The remaining reads were a mixture of microbes (8.1%), primate (1.7%), and other eutherian (0.5%) transcripts.
(TIF)
