# Supplementary figures and images for: Limited Genetic Diversity Preceded Extinction of the Tasmanian Tiger
Source: PLoS One. 2012 Apr 18;7(4):e35433. doi: 10.1371/journal.pone.0035433 (PMC3329426; doi:10.1371/journal.pone.0035433)

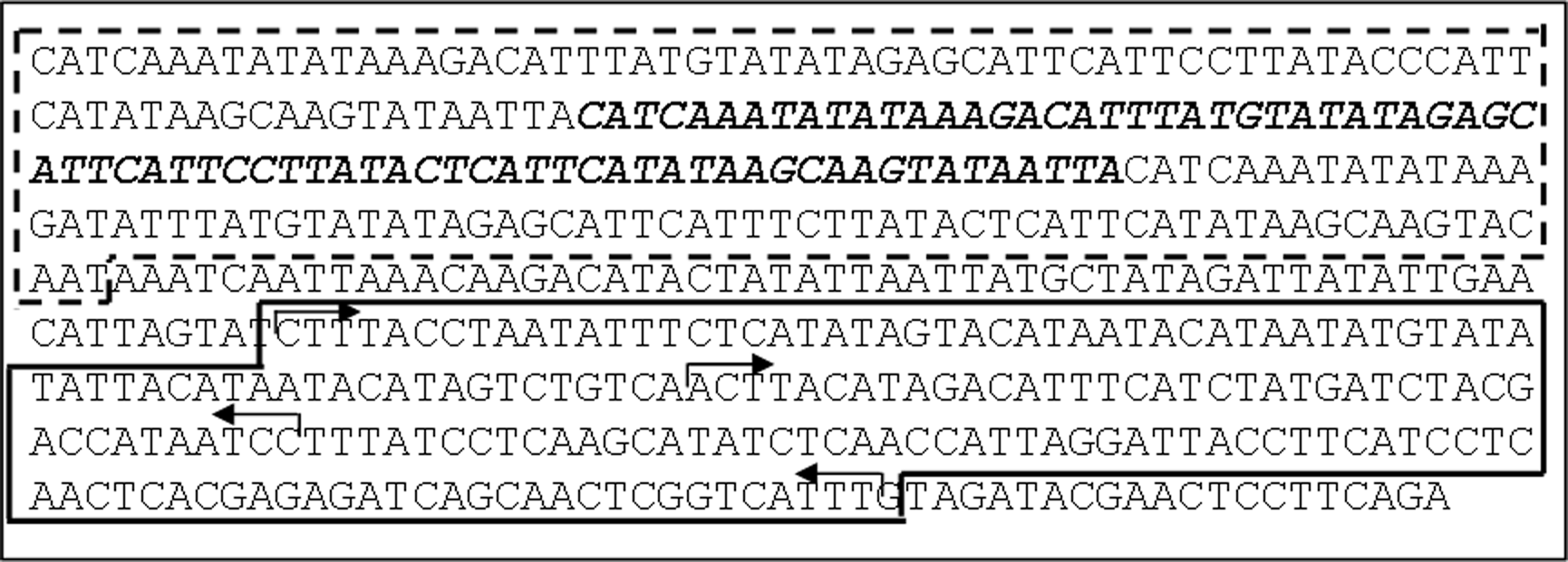

Supplement: Figure S1 — Position of repetitive element and the amplified region within the thylacine mtDNA CR-1. The thylacine CR-1 contains a repetitive sequence of 72–75 nucleotides in triplicate. This region was avoided as the organization of small <100 bp fragments could not determined. Instead, we targeted a 187 bp region after the repeat (Broken line box: repetitive section; unbroken line box: sequenced section; Arrows: position of forward and reverse primers). (TIFF) [file pone.0035433.s001.tif]

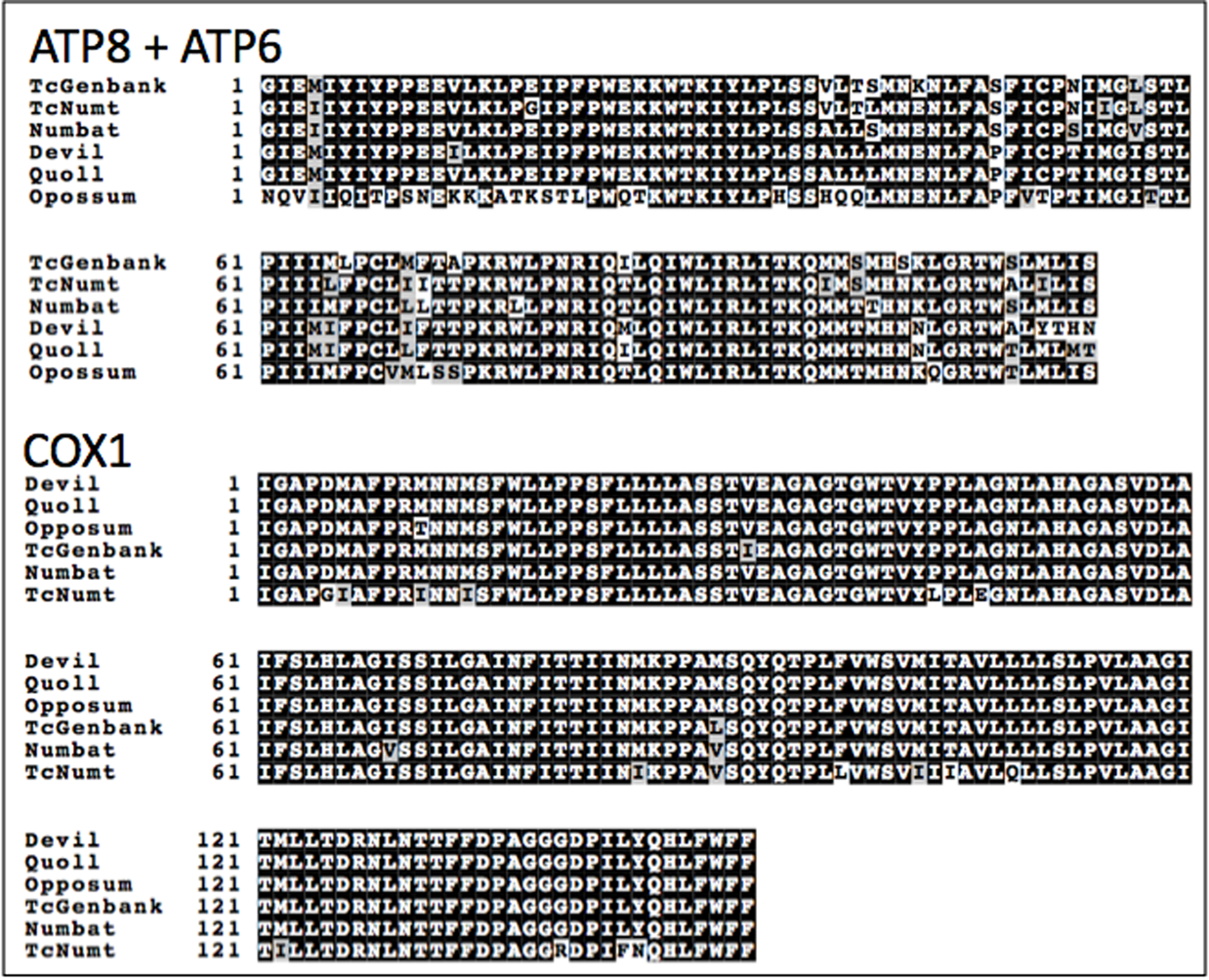

Supplement: Figure S2 — Amino acid alignment of the presumptive ATP8/ATP6 and COX1 NuMts relative to other marsupial homologs. Our presumptive NuMts for the ATP8/ATP6 and COX1 genes encoded 15 and 11 amino acid substitutions relative to the published thylacine homologs, respectively. The COX1 differences contrast much clearer than the ATP8/ATP6 differences as this gene is highly conserved in mammals. (TIF) [file pone.0035433.s002.tif]

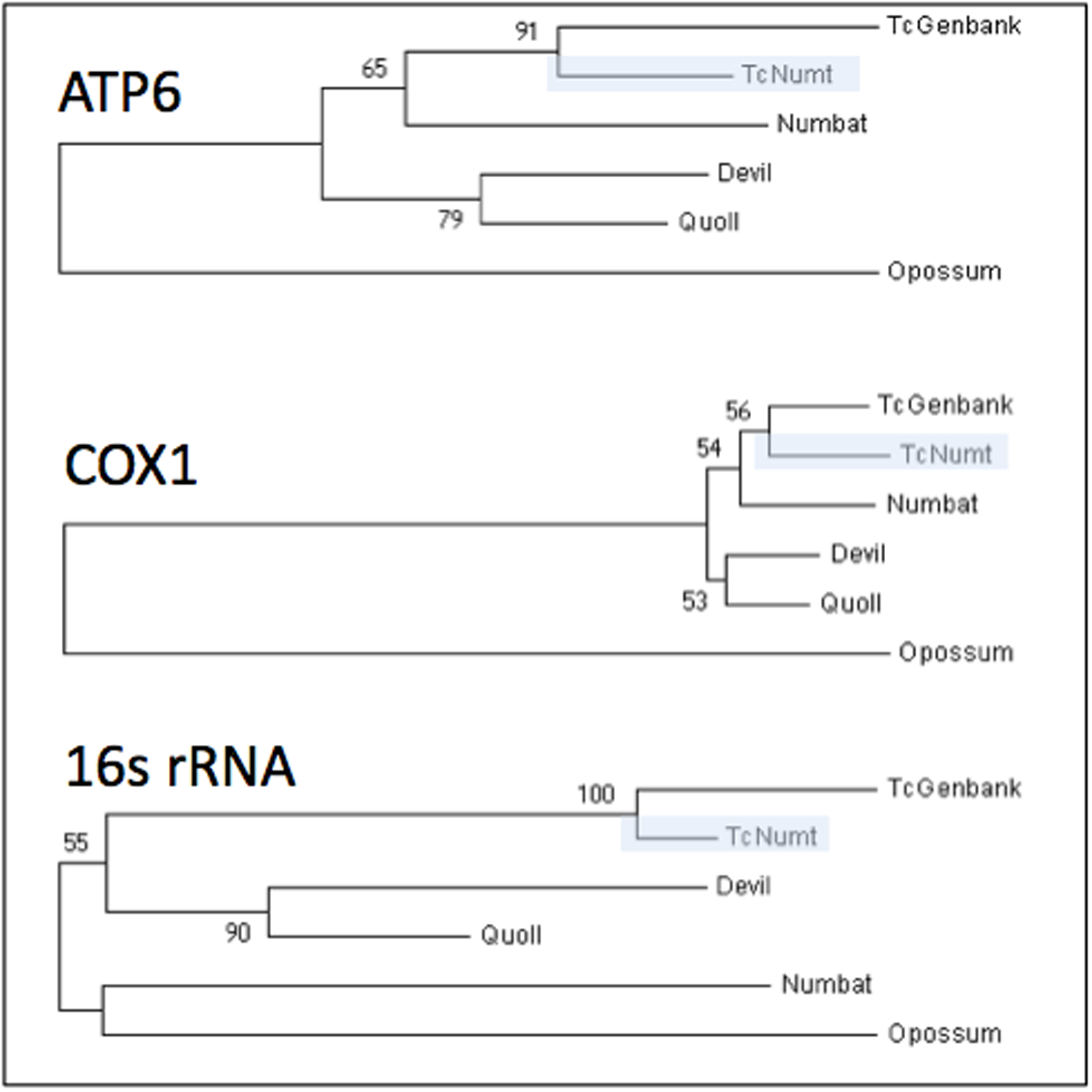

Supplement: Figure S3 — Phylogenetic relationship of the variable ATP8/ATP6 , COX1 and 16s rRNA nucleotide sequences. DNA sequence analysis of the variable thylacine sequences (TcNumt) grouped consistently with the sequences from the published thylacine mtDNA genome (TcGenbank) indicating that they likely inserted into the nuclear genome after the divergence of the thylacine from other marsupials. Trees represent the most conservative estimates of parsimony attained from using a combination of models (see materials and methods). There were no differences in the groupings for individual genes between models. (TIF) [file pone.0035433.s003.tif]

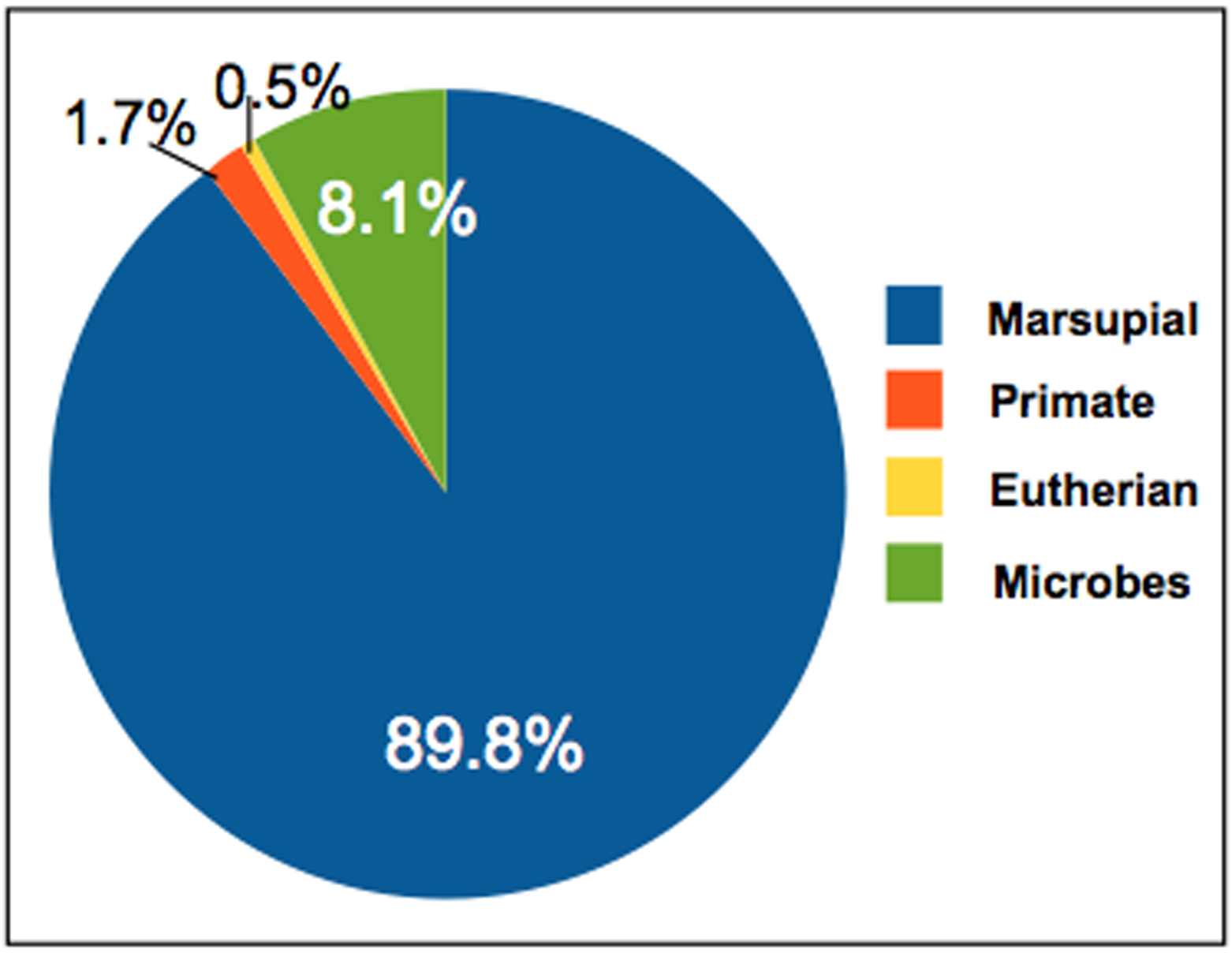

Supplement: Figure S4 — Quantity of transcripts derived from the thylacine 454 preparation. The 454 preparation was overwhelmingly composed of thylacine-specific transcripts, which made up 90% of the reads. The remaining reads were a mixture of microbes (8.1%), primate (1.7%), and other eutherian (0.5%) transcripts. (TIF) [file pone.0035433.s004.tif]
